# Histopathological Imaging–Environment Interactions in Cancer Modeling

**DOI:** 10.3390/cancers11040579

**Published:** 2019-04-24

**Authors:** Yaqing Xu, Tingyan Zhong, Mengyun Wu, Shuangge Ma

**Affiliations:** 1 Department of Biostatistics, Yale University, New Haven, CT 06520, USA; yaqing.xu@yale.edu; 2 SJTU-Yale Joint Center for Biostatistics, Department of Bioinformatics and Biostatistics, School of Life Sciences and Biotechnology, Shanghai Jiao Tong University, Shanghai 200240, China; tyzhong@sjtu.edu.cn; 3 School of Statistics and Management, Shanghai University of Finance and Economics, Shanghai 200433, China

**Keywords:** cancer modeling, interaction, histopathological imaging, clinical/environmental factors

## Abstract

Histopathological imaging has been routinely conducted in cancer diagnosis and recently used for modeling other cancer outcomes/phenotypes such as prognosis. Clinical/environmental factors have long been extensively used in cancer modeling. However, there is still a lack of study exploring possible interactions of histopathological imaging features and clinical/environmental risk factors in cancer modeling. In this article, we explore such a possibility and conduct both marginal and joint interaction analysis. Novel statistical methods, which are “borrowed” from gene–environment interaction analysis, are employed. Analysis of The Cancer Genome Atlas (TCGA) lung adenocarcinoma (LUAD) data is conducted. More specifically, we examine a biomarker of lung function as well as overall survival. Possible interaction effects are identified. Overall, this study can suggest an alternative way of cancer modeling that innovatively combines histopathological imaging and clinical/environmental data.

## 1. Introduction

Cancer is extremely complex. Extensive statistical investigations have been conducted, modeling various cancer outcomes/phenotypes. A long array of measurements from different domains have been used in cancer modeling, including clinical/environmental factors, socioeconomic factors, omics (genetic, genomic, epigenetic, proteomic, etc.) measurements, histopathological imaging features, and others. However, none of the existing models is completely satisfactory, and it remains a challenging task to develop new ways of cancer modeling.

Imaging has been playing an irreplaceable role in cancer practice and research [1]. It is routine for radiologists to use Computed Tomography (CT), Magnetic Resonance Imaging (MRI), Positron Emission Computed Tomography (PET), and other techniques to generate radiological images, which can inform the size, location, and other “macro” features of tumors [2]. Biopsies are ordered, and pathologists review the slides of representative sections of tissues to make definitive diagnosis. This procedure generates histopathological (diagnostic) images [3]. Through microscopically examining small pieces of tissues, more “micro” features of tumors are obtained. Histopathological images have been used as the gold standard for diagnosis. More recently, histopathological imaging features have also been used to model other cancer outcomes/phenotypes. For example, in [4], they were used for predicting the prognosis of estrogen receptor-negative breast cancer, and a multivariate Cox regression was adopted. In [5], histopathological imaging features were used in a k-nearest neighbor classifier to assign images into different groups of Gleason tumor grading for prostate cancer patients.

With the complexity of cancer, a single domain of measurement is insufficient, and measurements from multiple sources are needed in modeling [6]. In the literature, histopathological imaging features and clinical/environmental risk factors have been combined in an additive manner for modeling cancer outcomes. In [7], for modeling lung cancer prognosis, clinical factors (including age, gender, cancer type, smoking history, and tumor stage) were combined with imaging features in a multivariate Cox regression model. This study and those alike have shown that combining the two sources of information are more informative than a single source. Our literature review suggests that most if not all of the existing studies have considered the additive effects of histopathological imaging features and clinical/environmental factors, and *studies that accommodate their interactions (referred to as “I–E” interactions, with “I” and “E” standing for imaging and clinical/environmental factors, in this study) are lacking*. Statistically, adding interactions when the main-effect models are not fully satisfactory is “normal”. Biologically speaking, incorporating such interactions have been partly motivated by the success of gene–environment (G–E) interactions. Specifically, in the literature, the biological rationale and practical success of G–E interactions have been well established [8]. Cancer is a genetic disease. Histopathological images reflect essential information on the histological organization and morphological characteristics of tumor cells and their surrounding tumor microenvironment, which are heavily regulated by tumors’ molecular features. As such, from G–E interactions, we may naturally derive I–E interactions. It is noted that I–E and G–E interaction analyses cannot replace each other. More specifically, not all genetic information is contained in imaging features, and histopathological features, as reflected in imaging data, are also affected by factors other than molecular changes.

This study has also been partly motivated by the ineffectiveness of techniques adopted in the existing studies. Histopathological images contain rich information, and the number of extracted features can be quite large, posing analytic challenges. This dimension problem is “brutally” handled in some studies. For example, in [9], the univariate Cox model was fit to each imaging feature, and those with the strongest marginal effects were selected. Such features were then used along with clinical characteristics, including age, gender, smoking status, and tumor stage, to construct the final prognostic model. When joint modeling is the ultimate goal, the aforementioned approach may miss truly important signals in the first step of screening. To accommodate the high dimensionality in joint modeling, penalization and other regularization techniques have been adopted. For example, in [10], the elastic net approach, which combines the Lasso and ridge penalties, was used along with Cox regression. With the differences between interactions and main effects, such methods cannot be directly applied to analysis that accommodates I–E interactions. There are also studies that use advanced deep learning techniques. For example, Bychkov and others [11] used the CNN (convolutional neural network) technique to predict colorectal cancer prognosis based on images of tumor tissue samples. Other examples also include [12,13]. Such deep learning techniques may excel in prediction, however, usually lack interpretations and also suffer from a lack of stability when sample size is small.

The main objective of this article is to explore accommodating I–E interactions in cancer modeling. Although the concept may seem simple, such an interaction analysis has not been conducted in the literature. The adopted statistical methods have been “borrowed” from G–E interaction analysis. With the connectedness between genetic and histopathological imaging features and parallelization of G–E and I–E interaction analysis, such a strategy is sensible. The proposed interaction analysis strategy and methods are demonstrated using the The Cancer Genome Atlas (TCGA) lung adenocarcinoma data. Overall, this study may suggest an alternative way of utilizing histopathological imaging data and modeling cancer more accurately.

## 2. Data

We demonstrate I–E interaction analysis using the TCGA lung cancer data. TCGA is a collective effort organized by lNational Cancer Institute (NCI) and has published comprehensive data, especially on outcomes/phenotypes, clinical/environmental measures, and histopathological images, for lung and other cancer types. Lung cancer is the leading cause of cancer death globally [14], and lung adenocarcinoma (LUAD) is the most common histological subtype and has posed increasing public concerns [15]. The TCGA LUAD data has been analyzed in multiple published studies, including [7,9], who analyzed histopathological images, and [16,17], who conducted analysis on clinical/environmental factors. Thus, it is of interest to “continue” these studies on main additive effects and further examine potential I–E interactions with the TCGA LUAD data. It also has the advantage of having a relatively larger sample size, which is critical to achieve meaningful findings. It is noted that the proposed analysis can be directly applied to data on other cancer types.

We acquire 541 whole slide histopathology images from the TCGA ldata portal [18]. To extract imaging features, we adopt the following pipeline developed by Luo and others [9]. First, as the size of the whole slide images, which is from 300 Mb up to 2 Gb with 110,000 × 70,000 pixels, is too huge to be analyzed directly, each image is cropped into sub-images with 500 × 500 pixels and saved as tiff image files using the Openslide Python library. Analyzing all the sub-images (more than 10 million image tiles in total) is still computationally unfeasible. Thus, twenty representative tiff sub-images that contain mostly (>50%) regions of interest are randomly selected as input for the following process. It is expected that the randomly selected sub-images are representative samples for the overall “population” of sub-images. Such cropping and random selection are common steps in whole slide image processing and widely adopted in published imaging studies [10,19,20,21]. It is noted that randomly selecting sub-images may lead to imaging features with very small differences (and so affect downstream analysis). However, as our main goal is cancer model building, as opposed to feature selection, such small differences may not be of major concern.

Second, we adopt *CellProfiler* [22], a platform designed for cell image processing and used in quite a few recent publications, to extract quantitative features from each sub-image. Specifically, image colors are separated based on hematoxylin and eosin staining, and converted to grayscale for extracting regional features. Next, cell nuclei are detected and segmented so that cell-level features can be specifically measured. Other features such as regional occupation, area fraction, and neighboring architecture are also captured. Irrelevant features such as file size and execution information are excluded from analysis. This procedure results in a total of 772 features which are categorized into the texture, geometry, and holistic groups. Specifically, the texture group contains Haralick, Gabor “wavelet”, and Granularity features, which are classic image processing features, measure the texture properties of cells and tissues, and have been examined in a large number of imaging studies. The geometry group contains features that describe the geometry properties (such as area, perimeter, and so on), and those extracted by Zernike moments. The holistic group contains holistic statistics that describe overall information, such as the total area, perimeter and number of nuclei, and nuclear staining area fraction.

Third, for each patient, the features of images are normalized using sample mean at the patient level. Missing values (with a missing rate lower than 20%) are imputed using sample medians.

For clinical/environmental risk factors, we consider age, American Joint Committee on Cancer tumor pathologic stage, tobacco smoking history indicator, and sex. These variables have been suggested as associated with multiple lung cancer outcomes/phenotypes, including those analyzed in this article [23]. In particular, Nordquis and others [24] found that the mean age at diagnosis of lung adenocarcinoma among never-smokers was significantly higher than that among current smokers, and the never-smokers with lung adenocarcinoma were predominantly female. Studies have shown that tobacco smoking is responsible for 90% of lung cancer [25], and has been identified as a negative prognostic factor for lung adenocarcinoma [26]. In addition, these factors have also been considered in G–E interaction analysis [27].

Multiple outcome variables have been analyzed in the literature [7]. In this article, we consider two important response variables: (a) FEV1: the reference value for the pre-bronchodilator forced expiratory volume in one second in percent. It is an important biomarker for lung capacity. It is continuously distributed, with mean 80.28 and interquartile range [67.00, 96.25]. Data is available for 132 subjects; and (b) overall survival, which is subject to right censoring. Data is available for 271 subjects, among whom 102 died during follow-up. The mean observed time is 27.47 months, with interquartile range [14.06, 35.00].

The adopted feature extraction process follows [9], where the extracted imaging features were used to predict lung cancer prognosis. Similar processes have also been adopted in other publications [10,19]. Different from limited histopathological features recognized visually by pathologists, CellProfiler extracted features are morphological features of tissue texture, cells, nuclei, and neighboring architecture. These features are extracted and measured by comprehensive computer algorithms, and are impossible to be assessed by human eyes. As demonstrated in [9], quantitative imaging features provide objective and rich information contained in images that can reveal hidden information to decode tumor development and progression in lung cancer. Following the literature [9,20,21], we adopt feature names automatically assigned by CellProfiler, as can be partly seen in Table 1, Table 2, Table 3 and Table 4. These names provide a brief description of the extracted information with the general form “Compartment_FeatureGroup_Feature_Channel_Parameters”. For example, features “AreaShape_MedianRadius” and “AreaShape_MaximumRadius” measure the median and maximum radius of the identified tissue, respectively. As in some recent studies [9,20,21], in this study, our goal is not to identify specific imaging features as markers and make biological interpretations. Instead, we aim to conduct better cancer modeling by incorporating I–E interactions. As such, although they may not have simple, explicit biological interpretations, these features are sensible for our analysis.

## 3. Methods

**Figure 1:**
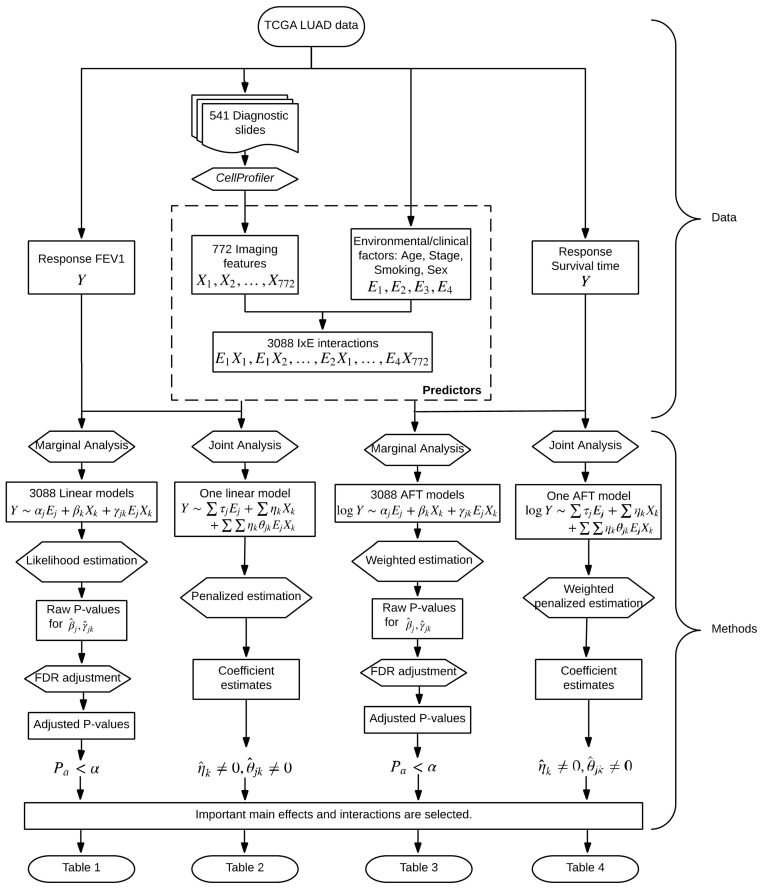
Flowchart of the I–E interaction analysis of The Cancer Genome Atlas (TCGA) lung adenocarcinoma (LUAD) data.

In parallel to G–E interaction analysis [28], we conduct two types of I–E interaction analysis, namely marginal and joint analysis. The overall flowchart of analysis is provided in Figure 1. In marginal analysis, one imaging feature, one clinical/environmental variable (or multiple such variables), and their interaction are analyzed at a time. In joint analysis, all imaging features, all clinical/environmental variables, and their interactions are analyzed in a single model. The two types of analysis have their own pros and cons and cannot replace each other. We refer to the literature [29,30] for more detailed discussions on the two types of analysis.

First, consider a continuous cancer outcome, which matches the FEV1 analysis. Denote *Y* as the length *N* vector of outcome, where *N* is the sample size. Denote **E** = [*E*_1_, ⋯, *E_J_*] as the *N* × *J* matrix of clinical/environmental variables, and **X** = [*X*_1_, ⋯, *X_K_*] as the *N* × *K* matrix of imaging features. As represented by the LUAD data, usually clinical/environmental variables are pre-selected and low-dimensional, and imaging features are high-dimensional.

### 3.1. Marginal Analysis

Detailed discussions of marginal G–E interaction analysis are available in [31] and other recent literature. The marginal I–E interaction analysis proceeds as follows. First, assume that *Y*, **E**, and **X** have been properly centered.

(a) For j=1,…,J and k=1,…,K, consider the linear regression model
  (1) $$Y=\alpha_{j}E_{j}+\beta_{k}X_{k}+\gamma_{jk}E_{j}X_{k}+\epsilon ,$$
  where $\alpha_{j}$ and $\beta_{k}$ respectively represent the main effects of the *j*th clinical/environmental factor and the *k*th imaging feature, $\gamma_{jk}$ is the interactive effect, and *ϵ* is the random error. A total of *J* × *K* models are built.
(b) As each model has a low dimension, estimates can be obtained using standard likelihood based approaches and existing software. *p*-values can be obtained accordingly.
(c) Interactions (and main effects) with small *p*-values are identified as important. When more definitive conclusions are needed, the false discovery rate (FDR) or Bonferroni approach can be applied.

It is noted that, in Step (a), one clinical/environmental variable is analyzed in each model, which follows [31]. It is also possible to accommodate all clinical/environmental variables in each model. In Step (c), discoveries can be made on interactions only or interactions and main effects combined. Advantages of marginal analysis include its computational simplicity and stability. On the negative side, with the complexity of cancer, an outcome/phenotype is usually associated with multiple imaging features and clinical/environmental variables. As such, each marginal model can be “mis-specified” or “suboptimal”. In addition, there is a lack of attention to the differences between interactions and main effects.

### 3.2. Joint Analysis

Joint analysis can tackle some limitations of marginal analysis, and is getting increasingly popular in statistical and bioinformatics literature. It proceeds as follows.

(a) Consider the joint model
  (2) $$Y=\sum_{j=1}^{J}\tau_{j}E_{j}+\sum_{k=1}^{K}\eta_{k}X_{k}+\sum_{j=1}^{J}\sum_{k=1}^{K}\eta_{k}\theta_{jk}E_{j}X_{k}+\epsilon ,$$
  where $\tau_{j}$ and $\eta_{k}$ are the main effects of the *j*th environmental factor and the *k*th imaging feature, respectively, and the product of $\eta_{k}$ and $\theta_{jk}$ corresponds to the interaction.
(b) For estimation, consider the Lasso penalization
  (3) $$\min_{\eta_{k},\theta_{jk}}||Y-{f\left(E,X\right)||}^{2}+\lambda_{1}\sum_{k}|\eta_{k}|+\lambda_{2}\sum_{j}\sum_{k}\left|\theta_{jk}\right|,$$
  where $f\left(E,X\right)=\sum_{j}\tau_{j}E_{j}+\sum_{k}\eta_{k}X_{k}+\sum_{j}\sum_{k}\eta_{k}\theta_{jk}E_{j}X_{k}$, and $\lambda_{1},\lambda_{2}>0$ are tuning parameters. In numerical study, we select the tuning parameters using the extended Bayesian information criterion [32].
(c) Interactions (and main effects) with nonzero estimates are identified as being associated with the outcome.

### 3.3. Accommodating Survival Outcomes

Consider cancer survival. Denote *T* as the *N*-vector of survival times. Below, we describe joint analysis, and marginal analysis can be conducted accordingly. We adopt the AFT (accelerated failure time) model, under which

(4) $$\log\left(T\right)=\sum_{j=1}^{J}\tau_{j}E_{j}+\sum_{k=1}^{K}\eta_{k}X_{k}+\sum_{j=1}^{J}\sum_{k=1}^{K}\eta_{k}\theta_{jk}E_{j}X_{k}+\epsilon ,$$

where notations have similar implications as in the above section. With high-dimensional data, the AFT model has been widely adopted because of its lucid interpretation and more importantly computational simplicity [33]. Under right censoring, denote *C* as the *N*-vector of censoring times, *Y* = log(min(*T*, *C*)), and *δ* = *I*(*T* ≤ *C*), where operations are taken component-wise. To accommodate censoring, a weighted approach is adopted. Assume that data have been sorted according to *Y_i_*’s from the smallest to the largest. The Kaplan–Meier weights can be computed as $w_{1}=\frac{\delta_{1}}{N}$, $w_{i}=\frac{\delta_{i}}{N-i+1}\prod_{j=1}^{i-1}{\left(\frac{N-j}{N-j+1}\right)}^{\delta_{j}}$, i=2,…,N. Similar to Equation (3), consider the penalized estimation

(5) $$\min_{\eta_{k},\theta_{jk}}\left|\right|\sqrt{w}\times {\left(Y-f\left(E,X\right)\right)||}^{2}+\lambda_{1}\sum_{k}|\eta_{k}|+\lambda_{2}\sum_{j}\sum_{k}\left|\theta_{jk}\right|,$$

where the square root and multiplication are taken component-wise. Interpretations and other operations are the same as for continuous outcomes.

In joint analysis, the most prominent challenge is the high dimensionality. Here, the penalization technique is adopted, which can simultaneously accommodate high dimensionality and identify relevant interactions/main effects. Another feature of this analysis that is worth highlighting is that it respects the “main effects, interactions” hierarchy. That is, if an I–E interaction is identified, the corresponding main imaging feature effect is automatically identified. It has been suggested that, statistically and biologically, it is critical to respect this hierarchy [34]. We refer to the literature [35,36] for alternative penalization and other joint interaction analysis methods. Compared to marginal analysis, joint analysis can be computationally more challenging, and well-developed software packages are still limited. In addition, the analysis results can be less stable.

The proposed analysis can be effectively realized. To facilitate data analysis within and beyond this study, we have developed R code and made it publicly available at www.github.com/shuanggema.

## 4. Results

### 4.1. Analysis of FEV1

#### 4.1.1. Marginal Analysis

After the FDR adjustment, none of the main effects or interactions are statistically significant. In Table 1, we present the main effects and interactions with the smallest (unadjusted) *p*-values. The top ranked main effects are from the Geometry and Texture groups, and the top ranked interactions are from the Geometry group and with sex.

Based on the analysis results, we conduct a power calculation. First, assume the current levels of estimated effects and their variations. Then, with a sample size of 224, the top ranked I–E interactions can be identified as significant with target FDR 0.1. Second, consider the current sample size and levels of variations. Then, an effect of −0.35 can be identified as significant with target FDR 0.1.

For comparison, we conduct the analysis of main effects (without interactions). The top eight main effects (with the smallest *p*-values) have four overlaps with those in Table 1, suggesting that accommodating interactions can lead to different findings.

#### 4.1.2. Joint Analysis

The analysis results are provided in Table 2. A total of 11 imaging features are identified, representing the Geometry and Texture groups. A total of 11 interactions are identified, with all four clinical/environmental variables.

For comparison, we consider the joint model with all clinical/environmental variables and imaging features but no interactions. Lasso penalization is applied for selection and estimation. A total of eight imaging features are identified, with one overlapping with those in Table 2. We further compute the RV coefficient, which may more objectively quantify the amount of “overlapping information” between two analyses. Specifically, it measures the “correlation” between two data matrices of important effects identified by two different approaches, with a larger value indicating higher similarity. The RV coefficient is 0.24, suggesting a mild level of overlapping.

A significant advantage of joint analysis is that it can lead to a predictive model for the outcome variable. We conduct the evaluation of prediction based on a resampling procedure, which may provide support to the validity of analysis. Specifically, we split data into a training and a testing set, generate estimates using the training data, and make predictions for the testing set subjects. The PMSE (prediction mean squared error) is then computed. This procedure is repeated 100 times, and the mean PMSE is computed. The I–E interaction model has a mean PMSE of 0.84, whereas the main-effect-only model has a mean PMSE of 1.12. This significant improvement suggests the benefit of accommodating interactions.

### 4.2. Analysis of Overall Survival

#### 4.2.1. Marginal Analysis

The analysis results are provided in Table 3, where we present estimates, raw *p*-values, as well as the FDR adjusted *p*-values. Three imaging features from the Holistic group have the FDR adjusted *p*-values < 0.1. In addition, 36 imaging features from the Geometry group and 24 features from the Texture group are identified as having interactions with Smoking, the most important environmental factor for lung cancer. Compared to the above analysis, more “signals” are identified. Note that the effective sample size is smaller than that above. As such, the smaller *p*-values are likely to be caused by stronger signals.

For comparison, we conduct the analysis of main effects. One imaging feature is identified as having FDR adjusted *p*-value < 0.1, which is also identified in Table 3. With the complexity of lung cancer prognosis, the interaction analysis, which identifies more effects, can be more sensible.

#### 4.2.2. Joint Analysis

The analysis results are provided in Table 4. A total of 31 imaging features are identified, representing the three feature groups. Two imaging features are identified as interacting with two and four clinical/environmental variables, respectively.

**Figure 2:**
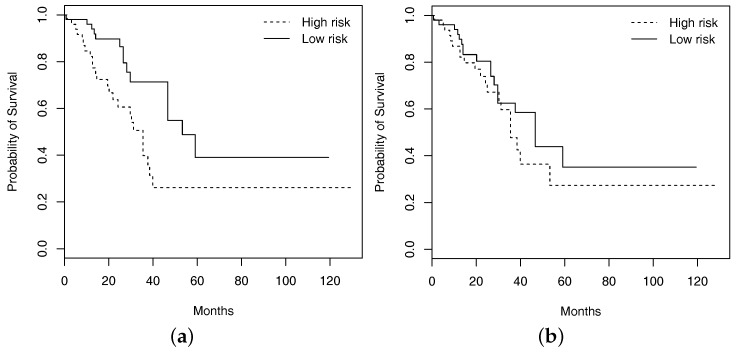
Kaplan–Meier curves of high and low risk groups identified by the approach that accommodates interactions ((**a**); logrank test *p*-value 0.007) and the one with main effects only ((**b**); logrank test *p*-value 0.320).

The analysis of main effects is conducted using the Lasso penalization. A total of two imaging features are identified, with one overlapping with those in Table 4. The RV coefficient is computed as 0.40, representing a moderate level of overlapping. As with FEV1, prediction evaluation is also conducted based on resampling. For the testing set, subjects are classified into low and high risk groups with equal sizes based on the predicted survival times, where subjects with predicted survival times larger than the median are classified into the low risk group. For one resampling of training and testing sets, in Figure 2, we plot the Kaplan–Meier curves estimated using the observed survival times for the predicted low and high risk groups, along with those generated under the additive main-effect model. Compared to the main-effect model, it is obvious that the two risk groups identified by the I–E interaction model have a much clearer separation of the survival functions, indicating better prediction performance. To be more rigorous, we further conduct a logrank test, which is a nonparametric test for comparing the survival distributions of two subject groups. With 100 resamplings, the average logrank statistics are 7.28 (I–E interaction model, *p*-value = 0.007) and 0.99 (main-effect model, *p*-value = 0.320), respectively. The superior prediction performance of the I–E interaction models suggests that incorporating interactions can lead to clinically more powerful models, justifying the value of the proposed analysis.

### 4.3. Simulation

Comparatively, joint analysis is newer and has been less conducted. To gain more insights into the validity of findings from our joint interaction analysis, we conduct a set of data-based simulation. Specifically, the observed imaging features and clinical/environmental factors are used. To generate variations across simulation replicates, we use resampling, with sample sizes set as 200. The “signals” and their levels are set as those in Table 2 and Table 4, respectively. For both the continuous and (log) survival outcomes, we generate random errors from *N*(0, 1). For the survival setting, we generate the censoring times from randomly sampling the observed. The Lasso-based penalization approach is then applied, with tuning parameters selected using the extended Bayesian information criterion (BIC) approach. To evaluate identification, TP (true positive) and FP (false positive) values are computed. Summary statistics are computed based on 100 replicates. Under the continuous outcome setting, there are 11 true main effects and 11 I–E interactions. For main effects, the TP and FP values are 9.75 (1.65) and 3.15 (1.39), respectively, where numbers in “()” are standard deviations. For interactions, the TP and FP values are 7.35 (0.99) and 0.05 (0.22), respectively. Under the censored survival outcome setting, there are 31 true main effects and 6 I–E interactions. For main effects, the TP and FP values are 24.41 (3.98) and 13.90 (2.47), respectively. For interactions, the TP and FP values are 3.24 (0.21) and 0.24 (0.12), respectively. Overall, at the estimated signal levels and with the observed feature distributions, the joint analysis is capable of identifying the majority of true interactions and main effects, with a moderate number of false discoveries. This provides a high level of confidence to the joint interaction analysis.

## 5. Discussion

Histopathological imaging analysis has been routine in cancer diagnosis, and recently, its application in the analysis of cancer biomarkers, outcomes, and phenotypes has been explored. This study has taken a natural next step and conducted the imaging-environment interaction analysis. Statistically and biologically speaking, the analysis has been partly motivated by G–E interaction analysis. It is noted that the statistical methods themselves have been almost fully “translated” from G–E interaction analysis. As I–E interaction analysis has not been conducted in published cancer modeling studies, it is sensible to first employ well-developed methods, and in the future, methods that are more tailored to imaging data may be developed. We also note that in cancer modeling and other biomedical fields, it is not uncommon to apply methods well developed in one field to other new fields. The proposed I–E interaction analysis, especially joint analysis, may seem considerably more complex than some cancer modeling approaches. With the complexity of cancer, models with a few variables and simple statistical analysis are getting increasingly insufficient. Published studies have suggested that advanced statistical techniques and complex models are needed. Recent developments for lung cancer, including the elastic net-Cox analysis [10], deep convolutional neural network [13], and deep network based on convolutional and recurrent architectures [11], have comparable or higher levels of complexity compared to the proposed analysis. Artificial intelligence (AI) techniques, which have been recently used for cancer modeling in particular including the radiomics analysis of non-small-cell lung cancer [37,38], have even higher levels of complexity. We conjecture that such complexity will also be needed for future developments in cancer modeling using imaging data. The increasing complexity in cancer modeling seems to be an inevitable trend, and domain specific expertise is a must for such analysis.

We have analyzed the TCGA LUAD data with a continuous and a censored survival outcome. This choice has been motivated by the clinical importance of lung adenocarcinoma as well as data availability (a larger sample size). It is noted that the proposed analysis and R program will be directly applicable to the analysis of data on other cancer types. I–E interactions have been identified in both marginal and joint analysis, for both FEV1 and overall survival. There is one prominent difference between imaging and genetic/clinical data. With extensive investigations and functional experiments, the biological and biomedical implications of most clinical/environmental factors and genes are at least partially known. It is thus possible to evaluate whether G–E interactions are biologically sensible. The circumstance is significantly different for histopathological imaging features. The rationale and algorithms for feature extraction have been made clear in the developments of CellProfiler and other software. However, the identified features do not have lucid biological interpretations. As such, we are not able to objectively assess the biological implications of the findings in Table 1, Table 2, Table 3 and Table 4. It is noted that this limitation is also shared by recently published imaging studies [9,20,21], which have unambiguously demonstrated the great value of such imaging features in cancer modeling. It is also noted that imaging features derived from computer-aided pathological analysis have the unique advantage of being objective and comprehensive, and can reveal hidden information contained in histopathological images that cannot be recognized or assessed by pathologists. Our statistical evaluations, including the prediction evaluation and data-based simulation, can provide support to the analysis results to a great extent. In general, more investigations into the biological implications of the computer-program-extracted imaging features will be needed.

This study has suggested a new venue for cancer modeling. Although findings made on LUAD may not be applicable to other cancers, the analysis technique and R program will be broadly applicable. Following the flowchart in Figure 1 and detailed steps described in this article, and using the publicly available R program, cancer biostatisticians and clinicians should be able to carry out the proposed analysis with their own data. More specifically, with their own clinical/environmental and imaging data, they will be able to construct models for prognosis and other outcomes/phenotypes. Such models, as other cancer models (for example those using omics data), can be used to assist clinical decision making. Overall, this study may help advance the challenging field of cancer modeling.

## 6. Conclusions

Histopathological imaging data may harbor important information on cancer and has been recently used for modeling cancer clinical outcomes and phenotypes. This study has been the first to examine the interactions between imaging features and clinical/environmental risk factors in cancer modeling. Marginal and joint analysis approaches have been described. In the analysis of TCGA LUAD data, it has been shown that I–E interactions may be important for modeling FEV1 and overall survival. Overall, this study has suggested a new paradigm of cancer bioinformatics modeling.

## Figures and Tables

**Table 1:** Marginal analysis of the reference value for the pre-bronchodilator forced expiratory volume in one second in percent (FEV1): identified main effects and interactions, with raw *p*-values *P_r_*.

Feature Group	Feature Name		Estimate	*P_r_*
Geometry	AreaShape_Zernike_2_2	Main	0.270	0.002
Geometry	AreaShape_Zernike_5_3	Main	−0.319	0.001
Geometry	Mean_Identifyhemasub2_AreaShape_Zernike_9_9	Main	−0.259	0.004
Geometry	Median_Identifyhemasub2_AreaShape_Zernike_7_1	Main	−0.249	0.005
Geometry	Median_Identifyhemasub2_AreaShape_Zernike_8_6	Main	−0.272	0.003
Texture	StDev_Identifyeosinprimarycytoplasm_Texture_Correlation_maskosingray_3_01	Main	0.280	0.002
Geometry	StDev_Identifyhemasub2_AreaShape_Zernike_8_8	Main	−0.251	0.005
Geometry	StDev_Identifyhemasub2_AreaShape_Zernike_9_1	Main	−0.259	0.004
Geometry	StDev_Identifyhemasub2_AreaShape_Center_Y	Sex	0.291	0.002
Geometry	StDev_Identifyhemasub2_AreaShape_Zernike_8_2	Sex	0.304	0.001
Geometry	StDev_Identifyhemasub2_Location_Center_Y	Sex	0.294	0.002

**Table 2:** Joint analysis of FEV1: identified main effects and interactions.

Feature Group	Feature Name	Main	Age	Stage	Smoking	Sex
			−0.049	−0.052	−0.002	0.006
Geometry	AreaShape_Zernike_2_2	0.163	0.040	−0.014	−0.185	
Geometry	AreaShape_Zernike_5_3	−0.053				
Geometry	AreaShape_Zernike_6_0	−0.034				
Texture	Granularity_10_ImageAfterMath	0.137	0.110	−0.020		0.064
Geometry	Location_Center_X	0.002				
Geometry	Mean_Identifyeosinprimarycytoplasm_Location_Center_X	0.005				
Geometry	Median_Identifyhemasub2_AreaShape_Zernike_7_1	−0.127	−0.073		0.072	0.003
Geometry	StDev_Identifyhemasub2_AreaShape_Zernike_8_2	−0.170		−0.083		0.188
Texture	StDev_Identifyhemasub2_Granularity_6_ImageAfterMath	−0.029				
Texture	Texture_AngularSecondMoment_ImageAfterMath_3_00	−0.044				
Texture	Texture_AngularSecondMoment_ImageAfterMath_3_03	−0.010				

**Table 3:** Marginal analysis of overall survival: identified main effects and interactions, with raw *p*-values *P_r_* and false discovery rate (FDR) adjusted *p*-values *P_a_*.

Feature Group	Feature Name		Estimate	*P_r_*	*P_a_*
Holistic	Threshold_FinalThreshold_Identifyeosinprimarycytoplasm	Main	−0.301	0	0.095
Holistic	Threshold_OrigThreshold_Identifyeosinprimarycytoplasm	Main	−0.301	0	0.095
Holistic	Threshold_WeightedVariance_identifyhemaprimarynuclei	Main	−0.360	0	0.077
Geometry	AreaShape_Area	Smoking	0.253	0.004	0.078
Geometry	AreaShape_MaximumRadius	Smoking	0.266	0.004	0.074
Geometry	AreaShape_MeanRadius	Smoking	0.265	0.005	0.079
Geometry	AreaShape_MedianRadius	Smoking	0.266	0.005	0.079
Geometry	AreaShape_MinFeretDiameter	Smoking	0.257	0.003	0.073
Geometry	AreaShape_MinorAxisLength	Smoking	0.264	0.002	0.07
Geometry	AreaShape_Zernike_4_4	Smoking	−0.241	0.005	0.079
Geometry	AreaShape_Zernike_7_3	Smoking	−0.308	0	0.027
Geometry	AreaShape_Zernike_8_4	Smoking	−0.242	0.007	0.096
Geometry	AreaShape_Zernike_8_6	Smoking	−0.252	0.005	0.079
Geometry	AreaShape_Zernike_9_1	Smoking	−0.303	0	0.027
Texture	Granularity_13_ImageAfterMath.1	Smoking	−0.317	0.001	0.054
Texture	Mean_Identifyeosinprimarycytoplasm_Texture_Correlation_maskosingray_3_03	Smoking	0.232	0.005	0.079
Geometry	Mean_Identifyhemasub2_AreaShape_Area	Smoking	0.297	0.001	0.049
Geometry	Mean_Identifyhemasub2_AreaShape_MaximumRadius	Smoking	0.318	0.001	0.049
Geometry	Mean_Identifyhemasub2_AreaShape_MeanRadius	Smoking	0.318	0.001	0.049
Geometry	Mean_Identifyhemasub2_AreaShape_MedianRadius	Smoking	0.308	0.002	0.054
Geometry	Mean_Identifyhemasub2_AreaShape_MinFeretDiameter	Smoking	0.299	0.001	0.049
Geometry	Mean_Identifyhemasub2_AreaShape_MinorAxisLength	Smoking	0.310	0.001	0.045
Geometry	Mean_Identifyhemasub2_AreaShape_Zernike_4_4	Smoking	−0.263	0.003	0.07
Geometry	Mean_Identifyhemasub2_AreaShape_Zernike_5_1	Smoking	−0.268	0.002	0.07
Geometry	Mean_Identifyhemasub2_AreaShape_Zernike_8_2	Smoking	−0.277	0.003	0.073
Geometry	Mean_Identifyhemasub2_AreaShape_Zernike_8_8	Smoking	−0.290	0.003	0.073
Geometry	Mean_Identifyhemasub2_AreaShape_Zernike_9_1	Smoking	−0.226	0.004	0.074
Texture	Mean_Identifyhemasub2_Granularity_13_ImageAfterMath	Smoking	−0.325	0.001	0.054
Texture	Mean_Identifyhemasub2_Texture_Correlation_ImageAfterMath_3_01	Smoking	0.330	0	0.039
Texture	Mean_Identifyhemasub2_Texture_Correlation_ImageAfterMath_3_02	Smoking	0.297	0.002	0.07
Texture	Mean_Identifyhemasub2_Texture_Correlation_ImageAfterMath_3_03	Smoking	0.397	0	0.01
Texture	Mean_Identifyhemasub2_Texture_SumVariance_ImageAfterMath_3_02	Smoking	0.258	0.007	0.093
Texture	Median_Identifyeosinprimarycytoplasm_Texture_Correlation_maskosingray_3_03	Smoking	0.233	0.004	0.079
Geometry	Median_Identifyhemasub2_AreaShape_Area	Smoking	0.344	0	0.027
Geometry	Median_Identifyhemasub2_AreaShape_MaxFeretDiameter	Smoking	0.242	0.005	0.079
Geometry	Median_Identifyhemasub2_AreaShape_MaximumRadius	Smoking	0.323	0.001	0.049
Geometry	Median_Identifyhemasub2_AreaShape_MeanRadius	Smoking	0.323	0.001	0.049
Geometry	Median_Identifyhemasub2_AreaShape_MedianRadius	Smoking	0.266	0.005	0.079
Geometry	Median_Identifyhemasub2_AreaShape_MinFeretDiameter	Smoking	0.346	0	0.027
Geometry	Median_Identifyhemasub2_AreaShape_MinorAxisLength	Smoking	0.342	0	0.027
Geometry	Median_Identifyhemasub2_AreaShape_Perimeter	Smoking	0.247	0.006	0.085
Geometry	Median_Identifyhemasub2_AreaShape_Zernike_4_4	Smoking	−0.242	0.002	0.059
Geometry	Median_Identifyhemasub2_AreaShape_Zernike_5_1	Smoking	−0.256	0.003	0.073
Texture	Median_Identifyhemasub2_Granularity_13_ImageAfterMath	Smoking	−0.311	0.001	0.049
Texture	Median_Identifyhemasub2_Texture_Correlation_ImageAfterMath_3_01	Smoking	0.319	0.001	0.049
Texture	Median_Identifyhemasub2_Texture_Correlation_ImageAfterMath_3_02	Smoking	0.274	0.005	0.081
Texture	Median_Identifyhemasub2_Texture_Correlation_ImageAfterMath_3_03	Smoking	0.394	0	0.01
Texture	StDev_Identifyeosinprimarycytoplasm_Texture_SumAverage_maskosingray_3_00	Smoking	0.272	0.003	0.073
Texture	StDev_Identifyeosinprimarycytoplasm_Texture_SumAverage_maskosingray_3_01	Smoking	0.273	0.003	0.073
Texture	StDev_Identifyeosinprimarycytoplasm_Texture_SumAverage_maskosingray_3_02	Smoking	0.270	0.004	0.074
Texture	StDev_Identifyeosinprimarycytoplasm_Texture_SumAverage_maskosingray_3_03	Smoking	0.275	0.003	0.073
Geometry	StDev_identifyhemaprimarynuclei_Location_Center_Y	Smoking	−0.245	0.007	0.093
Geometry	StDev_Identifyhemasub2_AreaShape_Zernike_8_4	Smoking	−0.280	0.001	0.045
Geometry	StDev_Identifyhemasub2_AreaShape_Zernike_8_8	Smoking	−0.236	0.007	0.094
Texture	StDev_Identifyhemasub2_Texture_SumVariance_ImageAfterMath_3_01	Smoking	0.266	0.007	0.096
Texture	StDev_Identifyhemasub2_Texture_SumVariance_ImageAfterMath_3_02	Smoking	0.283	0.005	0.079
Texture	StDev_Identifyhemasub2_Texture_SumVariance_ImageAfterMath_3_03	Smoking	0.283	0.006	0.084
Geometry	StDev_identifytissueregion_Location_Center_Y	Smoking	−0.289	0.002	0.059
Texture	Texture_Correlation_ImageAfterMath_3_01	Smoking	0.252	0.004	0.078
Texture	Texture_Correlation_ImageAfterMath_3_03	Smoking	0.329	0	0.027
Texture	Texture_Correlation_maskosingray_3_03	Smoking	0.237	0.004	0.074
Texture	Texture_Entropy_ImageAfterMath_3_01	Smoking	0.220	0.007	0.093
Texture	Texture_Entropy_ImageAfterMath_3_03	Smoking	0.233	0.004	0.074

**Table 4:** Joint analysis of overall survival: identified main effects and interactions.

Feature Group	Feature Name	Main	Age	Stage	Smoking	Sex
			−0.024	−0.317	−0.038	−0.088
Geometry	AreaShape_Zernike_6_0	−0.038				
Geometry	AreaShape_Zernike_6_4	−0.019				
Geometry	AreaShape_Zernike_6_6	0.052				
Geometry	AreaShape_Zernike_9_3	0.027				
Geometry	AreaShape_Zernike_9_5	0.153				
Texture	Granularity_10_ImageAfterMath.1	−0.033				
Texture	Granularity_9_ImageAfterMath	0.081				
Geometry	Mean_Identifyhemasub2_AreaShape_Center_X	0.002				
Geometry	Mean_Identifyhemasub2_AreaShape_Zernike_5_1	0.013				
Geometry	Mean_Identifyhemasub2_AreaShape_Zernike_6_2	−0.002				
Geometry	Mean_Identifyhemasub2_AreaShape_Zernike_6_4	−0.010				
Geometry	Mean_Identifyhemasub2_AreaShape_Zernike_9_9	−0.146				
Geometry	Mean_Identifyhemasub2_Location_Center_X	0.002				
Geometry	Mean_identifytissueregion_Location_Center_X	0.056				
Geometry	Median_Identifyeosinprimarycytoplasm_Location_Center_X	−0.071				
Geometry	Median_Identifyhemasub2_AreaShape_Zernike_4_0	0.023				
Geometry	Median_Identifyhemasub2_AreaShape_Zernike_7_3	0.083				
Geometry	Median_Identifyhemasub2_AreaShape_Zernike_8_4	−0.120				
Geometry	Median_Identifyhemasub2_AreaShape_Zernike_8_6	−0.098				
Geometry	Median_Identifyhemasub2_AreaShape_Zernike_9_1	−0.044				
Geometry	Median_identifytissueregion_Location_Center_Y	−0.063				
Holistic	Neighbors_SecondClosestDistance_Adjacent	−0.170		−0.072	0.002	
Geometry	StDev_Identifyeosinprimarycytoplasm_Location_Center_Y	0.095				
Texture	StDev_Identifyeosinprimarycytoplasm_Texture_DifferenceVariance_maskosingray_3_00	0.036				
Geometry	StDev_Identifyhemasub2_AreaShape_Orientation	−0.159				
Geometry	StDev_Identifyhemasub2_AreaShape_Zernike_8_8	−0.146				
Texture	StDev_Identifyhemasub2_Granularity_12_ImageAfterMath	−0.101				
Texture	StDev_Identifyhemasub2_Granularity_13_ImageAfterMath	0.327	0.130	0.072	−0.189	0.174
Texture	StDev_Identifyhemasub2_Granularity_9_ImageAfterMath	0.003				
Texture	StDev_Identifyhemasub2_Texture_SumVariance_ImageAfterMath_3_01	−0.034				
Geometry	StDev_identifytissueregion_Location_Center_Y	0.016				
